# Ultrasound-based comparative assessment of ipsilateral vs. contralateral axillary lymph nodes in breast cancer patients — a pilot study

**DOI:** 10.3389/fonc.2025.1658446

**Published:** 2025-11-28

**Authors:** Nicoleta Zenovia Antone, Roxana Pintican, Bogdan Fetica, Carmen Lisencu, Daria Marian, Andrei Roman, Adrian Trifa, Vlad Gâta, Maximilian Muntean, Bogdan Pop, Catalin Vlad, Patriciu Achimaş Cadariu

**Affiliations:** 1 Breast Cancer Center, Prof. Dr Ion Chiricuta Oncology Institute, Cluj-Napoca, Romania; 2 Department of Oncological Surgery and Oncological Gynecology, “Iuliu Hatieganu” University of Medicine and Pharmacy, Cluj-Napoca, Romania; 3 Department of Radiology, Prof. Dr Ion Chiricuta Oncology Institute, Cluj-Napoca, Romania; 4 Department of Radiology, “Iuliu Hatieganu” University of Medicine and Pharmacy, Cluj-Napoca, Romania; 5 Department of Pathology, Prof. Dr Ion Chiricuta Oncology Institute, Cluj-Napoca, Romania; 6 Discipline of Medical Genetics, Center for Research and Innovation in Personalized Medicine of Respiratory Diseases, “Victor Babes” University of Medicine and Pharmacy, Timisoara, Romania; 7 Center of Expertise on Rare Pulmonary Diseases, Clinical Hospital of Infectious Diseases and Pneumophysiology “Dr. Victor Babes”, Timisoara, Romania; 8 Department of Surgery, Prof. Dr Ion Chiricuta Oncology Institute, Cluj-Napoca, Romania

**Keywords:** axilla, lymph nodes, axillary metastasis, breast cancer metastasis, contralateral lymph nodes, contralateral axilla

## Abstract

**Background:**

Accurate axillary lymph node assessment is critical in breast cancer staging. This study aimed to evaluate whether ultrasound (US)-based measurements, particularly cortical thickness and comparison with contralateral lymph nodes, could improve diagnostic accuracy in detecting axillary metastasis.

**Methods:**

In this prospective study, 110 breast cancer patients underwent bilateral axillary US. Ipsilateral and contralateral lymph nodes were assessed for shape, size, cortical characteristics, and hilum preservation. Quantitative features were compared between metastatic and non-metastatic nodes. Diagnostic accuracy was evaluated using ROC analysis, and various cut-off values were tested.

**Results:**

Metastatic nodes showed significantly increased cortical thickness (median 7.5 mm vs. 2.1 mm, p<0.001), larger short axes, and irregular shapes. The cortical thickness of ipsilateral lymph nodes had an AUC of 0.967 with a 3.4 mm cut-off yielding 97.7% sensitivity and 89.1% specificity. Comparing ipsilateral and contralateral cortical thickness revealed a 2.05 mm difference as optimal (AUC 0.926, 86% sensitivity, 89.1% specificity). US-based assessment outperformed traditional imaging in accuracy (92.66% vs. 82.73%).

**Conclusions:**

Quantitative US assessment of axillary lymph nodes, especially cortical thickness and bilateral comparisons, enhances diagnostic accuracy in breast cancer. Integrating these measures may reduce unnecessary biopsies and improve staging efficiency.

## 1 Introduction

Survival outcomes in breast cancer are influenced by the extent of axillary lymph node involvement, which serves as a prognostic factor and directly influences treatment planning. Patients with node-negative breast cancer generally have better long-term survival, while increasing numbers of metastatic axillary nodes are associated with higher rates of recurrence and worse overall survival (1, 2). Accurate axillary staging is therefore essential to define optimal surgical and systemic treatment strategies and avoid under- or overtreatment. In the context of the evolving de-escalation of axillary surgery in clinically node-negative patients, the need for improved imaging techniques to reliably predict nodal status is increasingly recognized (3–5).

Various imaging modalities have been investigated to improve preoperative axillary staging. Ultrasound (US) is widely used due to its accessibility, affordability, and lack of ionizing radiation, with studies reporting sensitivities ranging from 49% to 64% and specificities between 78% and 92%, depending on the criteria used to define suspicious nodes (6, 7). Contrast-enhanced ultrasound (CEUS) has shown promise in enhancing the detection of subtle neovascularity in metastatic nodes, with sensitivity exceeding 80% in some cohorts, though its use remains limited to specialized centers (8, 9). Magnetic resonance imaging (MRI), particularly diffusion-weighted imaging, offers excellent soft-tissue contrast, with reported sensitivity between 67% and 84% and specificity up to 92%, but its routine application in axillary staging is limited by cost, availability, and variability in diagnostic performance (10–12). PET-CT offers high specificity for detecting nodal metastases, particularly in patients with more advanced disease, but its spatial resolution limits detection of small metastases (less than 1 cm), and its use in early-stage breast cancer remains controversial (13, 14).

Despite the range of available imaging options, none consistently achieve the accuracy required to fully replace sentinel lymph node biopsy (SLNB) in early-stage breast cancer. Moreover, imaging studies typically focus on the ipsilateral axilla, with little attention given to the potential diagnostic value of comparing nodes bilaterally within the same patient. Asymmetry between ipsilateral and contralateral nodes in cortical thickness, shape, and hilum preservation could provide important clues to metastatic involvement. By integrating comparative ultrasound analysis into the axillary evaluation process, we hypothesize that diagnostic accuracy could be improved, leading to better patient selection for biopsy and potentially reducing unnecessary invasive procedures.

Thus, the aim of our study is to evaluate the diagnostic and prognostic value of comparative US analysis of ipsilateral and contralateral axillary lymph nodes in breast cancer patients.

## 2 Methods

This prospective, unicentric study included consecutive breast cancer (BC) patients evaluated at our institution between October 2022 and October 2024. The study was IRB approved and all patients signed a written consent. Inclusion criteria comprised patients with histologically confirmed BC, without previous axillary surgery, who underwent bilateral axillary US assessment, prior to any treatment. We excluded patients with bilateral breast cancer, and patients with concurrent systemic diseases such as lymphoma, leukemia or autoimmune diseases (eg rheumatoid arthritis), as they may potentially affect the imaging appearance of the lymph nodes.

All US were performed using 2 US vendors (Samsung RS80 and GE 2022) with high-frequency linear transducers (7–12 MHz) by dedicated breast radiologists with at least 2 years of breast imaging experience. Lymph nodes were assessed for shape (oval, round, irregular), long and short axis, cortical thickness (value, focal or diffuse, homogeneous or heterogeneous with hyperechoic or cystic areas), presence of hyperechoic halo, and fatty hilum preservation. We considered suspicious lymph nodes, round, without fatty hilum, and diffuse or focal cortical thickening of 3mm. All suspicious nodes were US-guided biopsied using core needle biopsy with an automated 14G needle device, and confirmed on pathology. For the contralateral assessment, we selected the lymph node with the greatest cortical thickness for comparison, applying the same set of suspicious criteria as on the ipsilateral side All patients underwent surgery for the primary tumor, with concomitant sentinel lymph node biopsy (SNLB) or axillary dissection (ALND). Pathology data for the breast tumor included histologic type (invasive ductal, invasive lobular, etc.), tumor grade (Nottingham system), hormone receptor status (ER-positive if ≥1% nuclear staining; PR-positive if ≥1% nuclear staining), and HER2 status (positive if IHC 3+ or FISH amplified).

All the data from the study was analyzed using IBM SPSS Statistics 25 and illustrated using Microsoft Office Excel/Word 2021. Quantitative variables were tested for normal distribution using the Shapiro-Wilk Test and were expressed as means with standard deviations or medians with interquartile ranges. Independent quantitative variables with a non-parametric distribution were compared between groups using the Mann-Whitney U Test, while related samples were analyzed using Wilcoxon’s Test. Qualitative independent variables were presented as counts or percentages and tested between groups using Fisher’s Exact Test or Pearson’s Chi-Square Test. To further detail contingency table results, Z-tests with Bonferroni correction were applied, and McNemar’s Test was used for qualitative variables in related samples, with Bonferroni correction when necessary. ROC curves were employed to determine diagnostic performances and cut-offs for lymph nodes dimensions in predicting metastases, with performance assessed using AUC values and 95% confidence intervals. Optimal cut-offs were selected based on the highest Youden index, and corresponding sensitivities and specificities were calculated. Univariable and multivariable logistic regression models were used to estimate the effect of predictive variables on the presence of lymph nodes metastases, with effects quantified through odds ratios and 95% confidence intervals. These models were evaluated for significance, goodness-of-fit, and multicollinearity, with all statistical tests using a significance threshold of α=0.05.

## 3 Results

### 3.1 Study population

A total of 110 breast cancer patients were included, with a median age of 57 years (IQR: 49-65). Of these, 43 (39.1%) had histologically confirmed axillary lymph node metastases. Invasive ductal carcinoma of no special type (IDC-NST) was the most common histologic subtype, found in 77.3% of cases. Tumors were predominantly hormone receptor-positive (ER-positive in 85.8%, PR-positive in 73.6%), while HER2 positivity was detected in 16.8% of cases. Median tumor size was 22 mm (IQR 15–29 mm), and high proliferation (Ki-67% ≥20%) was present in 76.2% of tumors (**Table 1**).

**Table 1:** Clinical and pathological characteristics of the study population.

Parameter	Total	Without metastasis (N=67)	With metastasis (N=43)	P
Tumor size	22 (15-29)	20 (15-28)	23.5 (15.5-30.5)	0.407*
Histology type				
Invasive ductal carcinoma	85 (77.3%)	46 (68.7%)	39 (90.7%)	0.074**
Invasive lobular carcinoma	10 (9.1%)	9 (13.4%)	1 (2.3%)	
Ductal carcinoma in Situ	4 (3.6%)	3 (4.5%)	1 (2.3%)	
Encapsulated papillary carcinoma	4 (3.6%)	4 (6%)	0 (0%)	
Other	7 (6.4%)	5 (7.5%)	2 (4.7%)	
Nottingham grade				
Grade 1	16 (14.8%)	12 (17.9%)	4 (9.8%)	0.353**
Grade 2	55 (50.9%)	35 (52.2%)	20 (48.8%)	
Grade 3	37 (34.3%)	20 (29.9%)	17 (41.5%)	
IHC^+^ markers				
ER-positive	91 (85.8%)	59 (90.8%)	32 (78%)	0.088**
PR-positive	78 (73.6%)	52 (80%)	26 (63.4%)	0.072**
HER2-positive	18 (16.8%)	13 (19.7%)	5 (12.2%)	0.427**
Ki-67%	30 (18.5-50)	25 (15-40)	35 (20-50)	0.047*
Ki-67% ≥ 20%	77 (76.2%)	43 (69.4%)	34 (87.2%)	0.040***

*Mann-Whitney U Test, **Fisher’s Exact Test, ***Pearson Chi-Square Test ^+^Immunohistochemistry.

The bold highlight the sub-chapters and what is statistically significant in terms of numbers.

### 3.2 Ipsilateral axillary assessment

Metastatic lymph nodes were found more frequent with irregular shape (20.9% vs. 1.5%, p<0.001), and longer short axis (median 10 mm vs. 6.55 mm, p<0.001). Preservation of the fatty hilum was seen in 97% of non-metastatic nodes and only in 58.1% of metastatic cases (p<0.001). Patients with lymph node metastases had significantly thicker ipsilateral lymph nodes, with a median cortical thickness of 7.5 mm (IQR 4.8–12 mm) compared to 2.1 mm (IQR 1.6-2.82 mm) in non-metastatic cases (p<0.001) (**Table 2**).

**Table 2:** US characteristics of the ipsilateral lymph nodes proved to be metastatic.

Parameter	Total	Without metastasis (N=67)	With metastasis (N=43)	P
Lymph node shape				
Oval	86 (78.9%)	**59 (89.4%)**	**27 (62.8%)**	<0.001**
Round	11 (10.1%)	6 (9.1%)	5 (11.6%)	
Irregular	10 (9.2%)	**1 (1.5%)**	**9 (20.9%)**	
Lymph node size				
Long axis	17.4 (12-23.65)	15 (11.5-21.77)	19 (14.4-26)	0.031*
Short axis	7.5 (6-10.55)	6.55 (5.3-8.52)	10 (7.8-15)	<0.001*
Hyperechoic halo	2 (1.8%)	0 (0%)	2 (4.7%)	
Preserved fatty hilum	90 (81.8%)	65 (97%)	25 (58.1%)	<0.001**
Cortical appearance				
Cortical thickness	3.1 (2-6.55)	2.1 (1.6-2.82)	7.5 (4.8-12)	<0.001*
Focal cortical thickness	10 (9.2%)	3 (4.5%)	7 (16.3%)	0.048**
Cortical echogenicity				
Homogenous - hypoechoic	101 (92.7%)	62 (93.9%)	39 (90.7%)	0.657**
Heterogenous with hyperechoic areas	7 (6.4%)	4 (6.1%)	3 (7%)	
Heterogeneous with cystic areas	1 (0.9%)	0 (0%)	1 (2.3%)	

*Mann-Whitney U Test, **Fisher’s Exact Test.

The bold highlight the sub-chapters and what is statistically significant in terms of numbers.

### 3.3 Ipsilateral compared to contralateral axillary assessment

**Figure 1:**
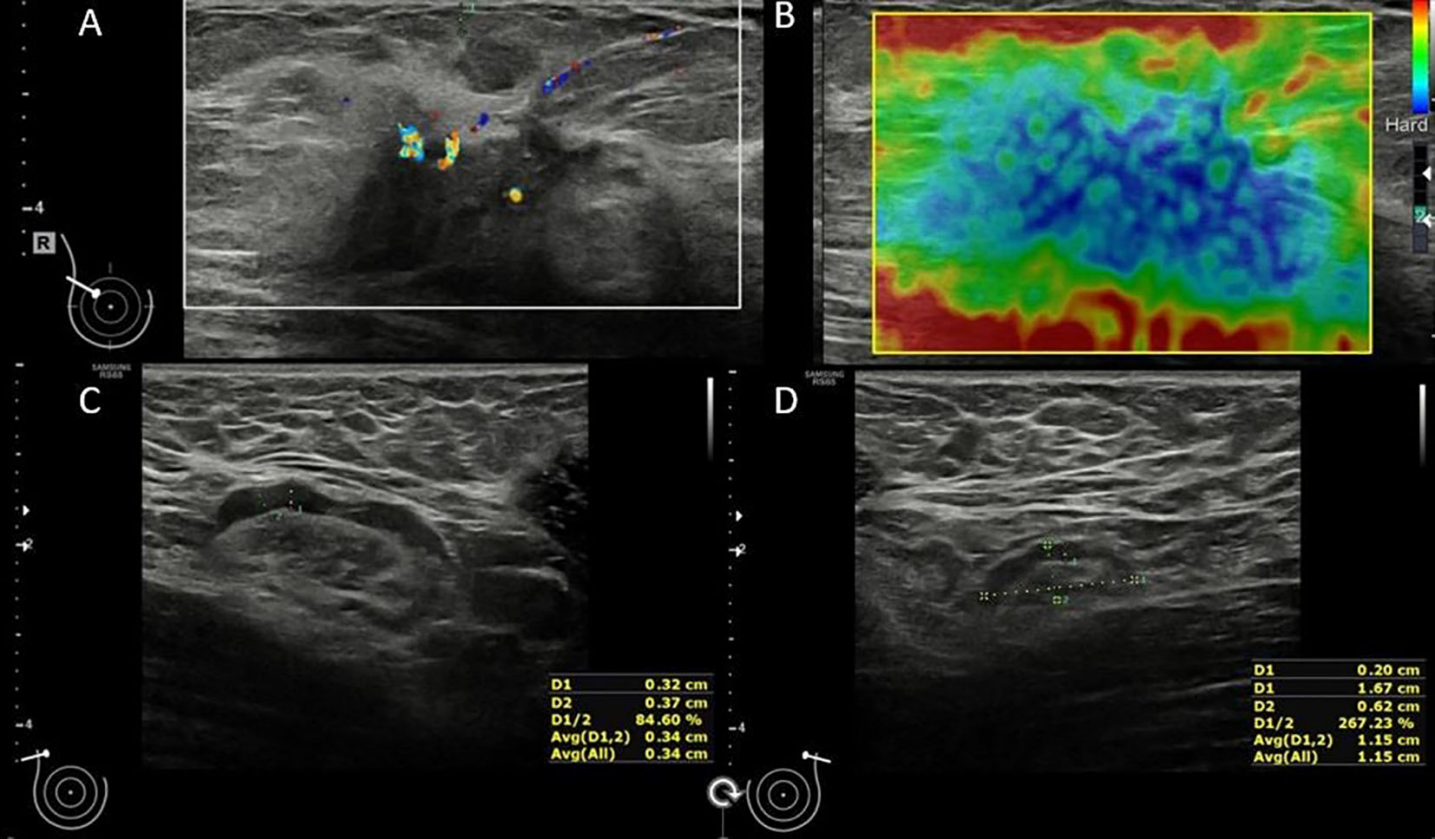
54 y old patient with right lobular breast cancer: In the upper-outer quadrant of the right breast, there is a hypoechoic mass, with internal vascularity **(A)** and stiff elastography appearance **(B)** – HP confirmed breast cancer; The ipsilateral lymph node **(C)** has preserved fatty hilum and a diffuse cortical thickness up to 3.2mm (HP confirmed metastasis); the contralateral lymph node shows a uniform cortex up to 2mm **(D)**.

The US showed the majority of the contralateral nodes with oval shape (97.2%), diffuse (98.1%) and homogenous - hypoechoic (97.1%) cortical appearance. The median for cortical thickness was 1.3 mm (IQR=1-2), long axis – 12 mm (IQR=9.8-18) and short axis – 6 mm (IQR=5-8.5) (**Figure 1**).

In patients with a confirmed axillary metastasis, the ipsilateral and contralateral lymph nodes showed significant morphological differences with larger nodes and thickened cortex in ipsilateral ones, compared to contralateral lymph nodes (all p-value < 0.05). The focal aspect was significantly more frequent in ipsilateral lymph nodeslymph nodes(9.5% vs. 1.9%, p=0.039), while cortical appearance did not differ significantly between ipsilateral and contralateral lymph nodeslymph nodes (**Table 3**).

**Table 3:** Differences of US characteristics between ipsilateral and contralateral axillary lymph nodes for patients with proved axillary metastasis.

Parameter	Ipsilateral	Contralateral	P-value
Lymph node shape			
Oval	27 (62.8%)	42 (97.7%)	<0.001***
Round	5 (11.6%)	1 (2.3%)	0.656***
Irregular	11 (25.5%)	0 (0%)	0.003***
Lymph node size (median in mm)			
Long axis	19 (14.4-26)	12 (9.2-17.7)	0.002*
Short axis	10 (7.8-15)	6.2 (4.6-8.9)	<0.001*
Hyperechoic halo	2 (4.7%)	–	
Cortical appearance			
Cortical thickness (median in mm)	7.5 (4.8-12)	1.4 (1.1-2.2)	<0.001*
Presence of focal cortical thickness	7 (17.1%)	0 (0%)	0.016**
Cortical echogenicity			
Homogenous - hypoechoic	38 (90.5%)	41 (97.6%)	0.500***
Heterogenous with hyperechoic areas	4 (9.3%)	2 (4.6%)	0.6
Heterogeneous with cystic areas	1 (2.4%)	–	

*Related-Samples Wilcoxon Signed Rank Test, **Related-Samples McNemar Test, ***Related-Samples McNemar Test with Bonferroni correction.

The bold highlight the sub-chapters and what is statistically significant in terms of numbers.

**Figure 2:**
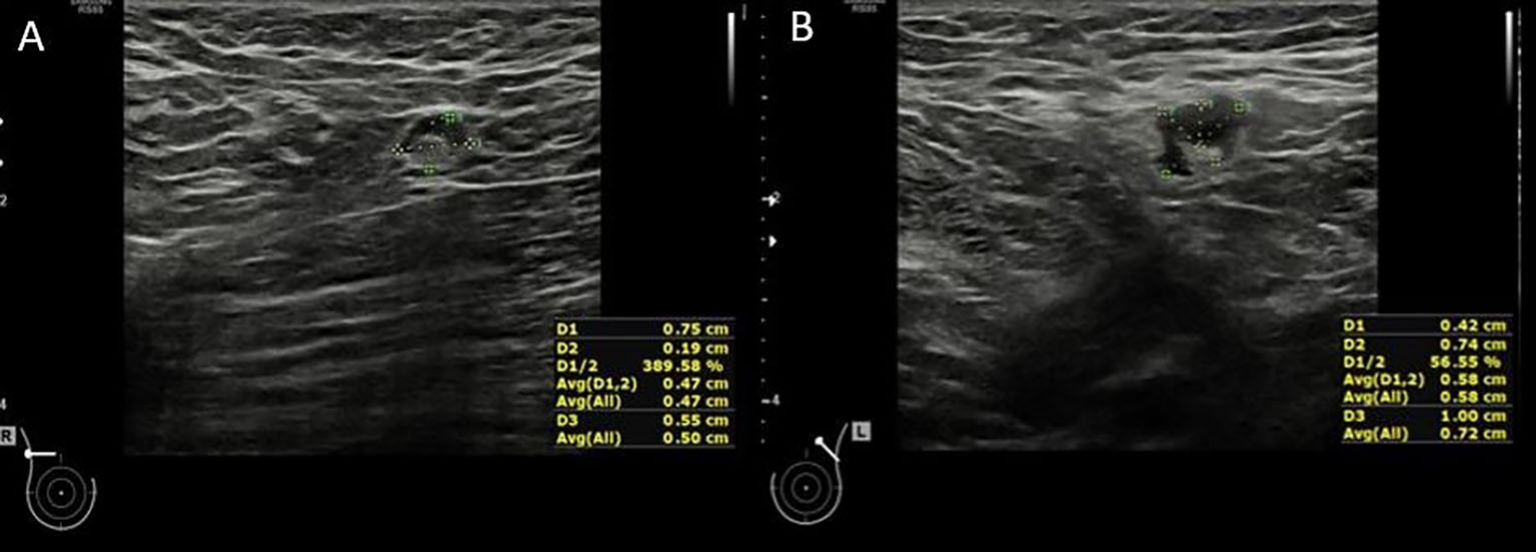
64 y old patient with left NST breast cancer; Axillary assessment is showing an ipsilateral abnormal lymph node, with preserved fatty hilum, but with cortical thickness >3mm, that was confirmed as metastasis **(A)**. The contralateral lymph node shows a fatty hilum and uniform cortex up to 1.9mm **(B)**. There was a cortical difference >2mm between the ipsilateral cortical thickness and the contralateral lymph node.

The same US features were found to be different in ipsilateral metastatic lymph nodes when compared to the contralateral ones (**Figure 2**). Patients with differing lymph node shapes between ipsilateral and contralateral sides were significantly more likely to have metastases (39.5% vs. 12.3%, p=0.002). Significant differences were observed in cortical thickness (median=5.9 mm, IQR=3.4–10.7 vs. median=0.7 mm, IQR=0.12–1.37, p<0.001) and short axis (p – value < 0.001) between ipsilateral and contralateral lymph nodes, with greater differences found in patients with metastases compared to those without. A similar trend was noted for the long axis, though it did not reach statistical significance (p=0.077). In contrast, differences in cortical echogenicity (p=1.000) between ipsilateral and contralateral lymph nodes was not statistically associated with the presence of metastases (**Table 4**).

**Table 4:** Comparison of the differences between ipsilateral and contralateral lymph nodes.

Parameter	Without axillary metastasis	With axillary metastasis	P
Lymph node shape			
Same shape	35 (81.4%)	26 (60.5%)	0.05**
Different shape	8 (12.3%)	17 (39.5%)	
Difference incortical thickness (mm)	0.7 (0.12-1.37)	5.9 (3.4-10.7)	<0.001*
Lymph node size (median in mm)	5 (7.8%)	7 (17.1%)	
Difference – Long axis	2.15 (-1.75 – 7.92)	7 (-1.2 – 14.5)	0.077*
Difference – Short axis	0.3 (-1 – 2)	3.3 (1.2 – 8.1)	<0.001*
Cortical echogenicity			
Similar appearance	39 (907%)	40 (93%)	1.000**
Different appearance	4 (9.3%)	3 (7%)	

*Mann-Whitney U Test, **Fisher’s Exact Test.

The bold highlight the sub-chapters and what is statistically significant in terms of numbers.

## 4 Diagnosis and prognosis analysis

The cortical length of ipsilateral lymph nodes showed an AUC of 0.967 (95% CI: 0.937–0.998, p<0.001). A cut-off value of 3.4 mm yielded a sensitivity of 97.7% and specificity of 89.1%, while a cut-off of 4.55 mm resulted in a sensitivity of 76.7% and specificity of 95.3%. The difference in cortical length between ipsilateral and contralateral lymph nodes had an AUC of 0.926 (95% CI: 0.870–0.983, p<0.001), with a 2.05 mm cut-off providing 86% sensitivity and 89.1% specificity. At a 3 mm cut-off, the sensitivity was 79.1% and specificity 95.3%, while a 3.95 mm cut-off resulted in 69.8% sensitivity and 96.9% specificity.

**Figure 3:**
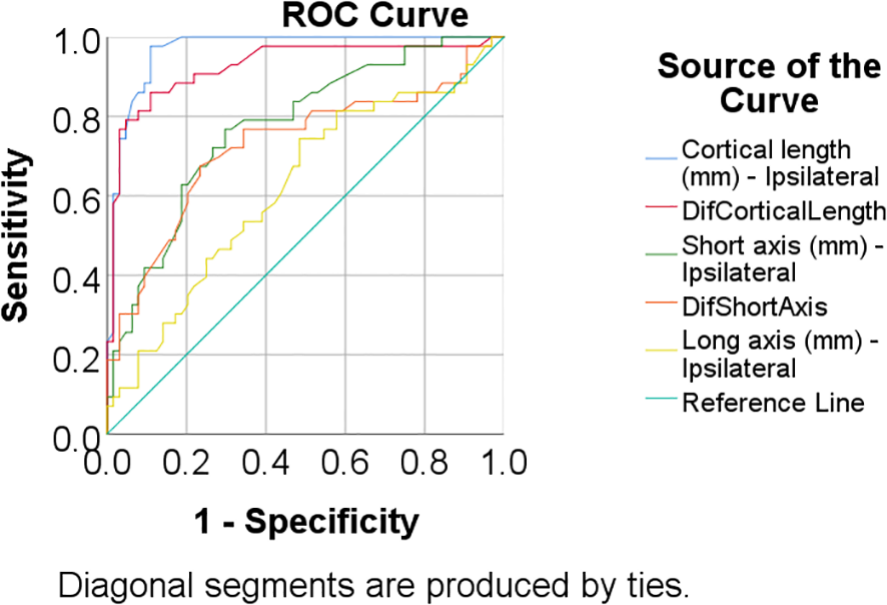
ROC curve used in the prediction of lymph node metastases based on US features.

The short axis of ipsilateral lymph nodes had an AUC of 0.776 (95% CI: 0.686–0.865, p<0.001), with a 7.7 mm cut-off yielding a sensitivity of 76.7% and specificity of 70.3%. The difference in short axis between ipsilateral and contralateral lymph nodes had an AUC of 0.729 (95% CI: 0.625–0.833, p<0.001), with a 2.05 mm cut-off providing 67.4% sensitivity and 76.6% specificity. The long axis of ipsilateral lymph nodes had an AUC of 0.623 (95% CI: 0.514–0.732, p=0.032), with a 15.35 mm cut-off yielding 74.4% sensitivity and 51.6% specificity (**Table 5**, **Figure 3**).

**Table 5:** ROC curves analyses used in estimation of lymph node metastases prediction.

Parameter	AUC (95% C.I.)	Std. Error	P	Cut-off	Se	Sp
Cortical thickness - ipsilateral	0.967 (0.937-0.998)	0.016	**<0.001**	3.4	97.7%	89.1%
Difference – Cortical thickness	0.926 (0.870-0.983)	0.029	**<0.001**	2.05	86%	89.1%
Short axis - ipsilateral	0.776 (0.686-0.865)	0.046	**<0.001**	7.7	76.7%	70.3%
Difference – Short axis	0.729 (0.625-0.833)	0.053	**<0.001**	2.05	67.4%	76.6%
Long axis – ipsilateral	0.623 (0.514-0.732)	0.056	**0.032**	15.35	74.4%	51.6%

The bold highlight the sub-chapters and what is statistically significant in terms of numbers.

Ultrasound correctly classified 37 metastatic lymph nodes and 54 benign lymph nodes. Furthermore, on US, there were 13 cases incorrectly labeled as metastatic (false positives) and 6 metastatic lymph nodes missed (false negatives). Overall, this resulted in a sensitivity of 86.05%, specificity of 80.6%, positive predictive value of 74%, negative predictive value of 90%, and an accuracy of 82.73% for detecting axillary metastasis.

The 3.4 mm cortical length cut-off for ipsilateral lymph nodeslymph nodes identified 59 true positives, 1 false positive, 7 false negatives, and 42 true negatives, with a sensitivity of 97.67%, specificity of 89.39%, positive predictive value of 85.71%, negative predictive value of 98.33%, and overall accuracy of 92.66%. The 2.05 mm cortical length difference cut-off identified 57 true positives, 6 false positives, 7 false negatives, and 36 true negatives, with a sensitivity of 85.71%, specificity of 89.06%, positive predictive value of 83.72%, negative predictive value of 90.48%, and overall accuracy of 87.74%. Forty-four patients (40%) underwent lymph nodeslymph nodes biopsy, with 11 (25%) testing negative for metastases. Among these, 6 patients (54.5%) had an ipsilateral cortical length < 3.4 mm or a cortical length difference < 2 mm.

## 5 Discussion

The results of this study reinforce axillary US as a key imaging modality for the prediction of lymph node metastasis in breast cancer patients. Comparative analysis between ipsilateral and contralateral axillary lymph nodes demonstrated significant morphological differences, with metastatic lymph nodes exhibiting larger dimensions, irregular shape, and increased cortical thickness. The cortical thickness of ipsilateral lymph nodes emerged as a strong predictor, with an AUC of 0.967 and a cut-off of 3.4 mm yielding a sensitivity of 97.7% and specificity of 89.1%. Additionally, the difference in cortical thickness between ipsilateral and contralateral nodes also demonstrated high predictive accuracy, with an AUC of 0.926 and an optimal cut-off of 2.05 mm providing 86% sensitivity and 89.1% specificity. These findings highlight the potential of using quantitative US measurements to refine lymph nodes assessment and improve diagnostic performance.

US remains the most widely used imaging modality for axillary staging in newly diagnosed breast cancer due to its accessibility, low cost, and ability to assess nodal morphology in real time. Multiple studies have shown that axillary US provides moderate to high diagnostic performance, with reported sensitivities of 60–80% and specificities of 80–95% (15–18). Recent advances in US technology have improved diagnostic accuracy in the contemporary literature (19), although this improvement has not been consistently observed in patients receiving neoadjuvant therapy (20). Despite the development and testing of several classification systems, substantial variability persists across countries and institutions (21). The NODE-RADS system has been introduced to enable a structured and harmonized assessment of lymph nodes on both US and MRI, and recent work has demonstrated comparable AUC performance in IDC and ILC, albeit with lower sensitivity in ILC (22–24). However, the system lacks validated biopsy thresholds, and its adoption in routine practice remains limited, as clinicians continue to favor simpler and more practical criteria such as cortical thickness cut-offs.

In our institution, a cortical thickness threshold of 3 mm is routinely applied, and the results of the present study are in line with previously published reports, yielding a sensitivity of 86.05%, specificity of 80.6%, positive predictive value of 74%, negative predictive value of 90%, and an overall accuracy of 82.73% for the identification of axillary metastases. When the cut-off for ipsilateral cortical thickness was increased to 3.4 mm, the overall accuracy improved to 92.66%, with a sensitivity of 97.67% and specificity of 89.39%. Likewise, applying a 2.05 mm inter-side cortical thickness difference cut-off resulted in an accuracy of 87.74%, with a sensitivity of 85.71% and specificity of 89.06%. These findings suggest that incorporation of quantitative criteria into routine axillary US assessment may improve diagnostic reliability and reduce both false-positive and false-negative classifications.

Compared with clinical examination alone, US provides substantially higher sensitivity for detecting axillary lymph node metastases (25, 26). However, when compared with advanced imaging such as MRI and PET/CT, the results are generally comparable. The existing literature on axillary imaging has focused almost exclusively on ipsilateral lymph node assessment (27–30), while reports addressing contralateral lymph nodes are scarce. When contralateral evaluation is mentioned, it is typically based on pathological examination and most often in the context of suspected or confirmed metastases (30, 31). More recently, interest has increased in the prognostic and therapeutic implications of synchronous contralateral axillary lymph node metastasis (CAM). Several studies have reported that patients with synchronous CAM demonstrate more favorable overall survival than those with other sites of distant metastasis, suggesting that CAM may represent a biologically distinct subgroup eligible for treatment with curative rather than palliative intent (30). In inflammatory breast cancer, CAM has been observed in approximately 8.3% of cases at presentation, with ultrasound identified as the most effective imaging modality for its detection. On this basis, recent authors recommend incorporating contralateral axillary US into the initial staging protocol for patients with inflammatory breast cancer (31).

To our knowledge, no prior study has systematically evaluated the contralateral axilla on imaging at initial breast cancer diagnosis. Our study addresses this significant gap by prospectively assessing the diagnostic appearance of contralateral axillary lymph nodes using US in newly diagnosed breast cancer patients. This approach could provide early insight into potential bilateral disease or atypical lymphatic drainage patterns and has the potential to inform staging and treatment planning in a way not previously explored in the imaging literature. US correctly classified lymph nodes as benign or malignant, with.

Our results highlight the potential to reduce unnecessary biopsies through the integration of bilateral cortical thickness assessments. In our cohort, a cortical thickness below 3.4 mm and a contralateral difference of only ~2 mm were not indicative of metastatic disease. These findings suggest that bilateral comparison primarily serves to reduce overdiagnosis in cases with mild and symmetrical cortical thickening, rather than increasing the sensitivity. Accordingly, contralateral assessment may help reduce false-positive interpretations and avoid unnecessary biopsies. Among the 44 patients who underwent lymph node biopsy, 11 (25%) had negative results for metastases. Of these, 6 patients (54.5%) had an ipsilateral cortical thickness below 3.4 mm or a cortical thickness difference of less than 2 mm, suggesting that in these cases, a negative biopsy could have been avoided. These findings suggest that incorporating cortical thickness measurements as part of a structured diagnostic workflow may assist in better patient selection for biopsy, thereby reducing the burden of invasive procedures without compromising diagnostic accuracy.

Besides classical imaging modalities, radiomics, artificial intelligence (AI), and machine learning are rapidly emerging as promising tools in breast imaging, including axillary lymph node assessment. Recent studies have shown that AI algorithms can enhance diagnostic accuracy, reduce inter-observer variability, and assist in risk stratification, particularly when integrated with clinical and pathological data (32–34). However, while initial results are encouraging, these technologies are still in early stages for routine axillary evaluation. Their performance is highly dependent on data quality, imaging standardization, and robust training across diverse populations. Thus, although AI and radiomics show strong potential, they are not yet ready to replace expert radiologist assessment in clinical practice, but rather serve as decision-support tools in well-validated workflows.

However, there are a few limitations: the study is unicentric, with a limited number of patients; other imaging modalities were not compared to US (such as breast MRI); future studies should further validate these cut-offs in larger, multi-institutional cohorts to assess their generalizability and clinical utility.

## 6 Conclusion

Quantitative US-based assessment of axillary lymph nodes, particularly cortical thickness measurements and comparative ipsilateral-contralateral analysis, enhances diagnostic accuracy in detecting metastatic involvement. The integration of objective measurement criteria, such as cortical thickness cut-offs, has the potential to refine patient selection for biopsy and reduce unnecessary invasive procedures. Further validation in larger cohorts is warranted to establish these criteria as standard practice in axillary staging for breast cancer patients.

## Data Availability

The original contributions presented in the study are included in the article/supplementary material, further inquiries can be directed to the corresponding author/s.
